# Hepatocellular Carcinoma in Africa: Challenges and Opportunities

**DOI:** 10.3389/fmed.2022.899420

**Published:** 2022-06-24

**Authors:** Mohamed El-Kassas, Mohamed Elbadry

**Affiliations:** Department of Endemic Medicine, Faculty of Medicine, Helwan University, Cairo, Egypt

**Keywords:** Africa, epidemiology, hepatocellular carcinoma (HCC), cancer, hepatitis C virus, hepatitis B

## Introduction

The African population accounts for 12% of the global population. Most of them live in the sub-Saharan Africa (SSA), where most inhabitants are Blacks. On the other hand, North Africa's inhabitants are Mediterranean rather than African in terms of the race, customs, and cultural background. That is why many of the distinctive features of cancers in Africa belong to SSA (1).

According to the latest Global Cancer Statistics (GLOBOCAN 2020), liver cancer is the 6th most common cancer, with more than 900,000 estimated annual new cases (4.7%). Its risk factors are well-known; however, it accounts for 8.3% of deaths of all cancers globally, being the third leading cause of worldwide cancer death (2). It is estimated that, by 2025, more than one million individuals will have liver cancer annually (3). Hepatocellular carcinoma (HCC) accounts for 75–85% of the liver cancer (PLC) (4).

## Epidemiology of HCC in Africa

**Figure 1:**
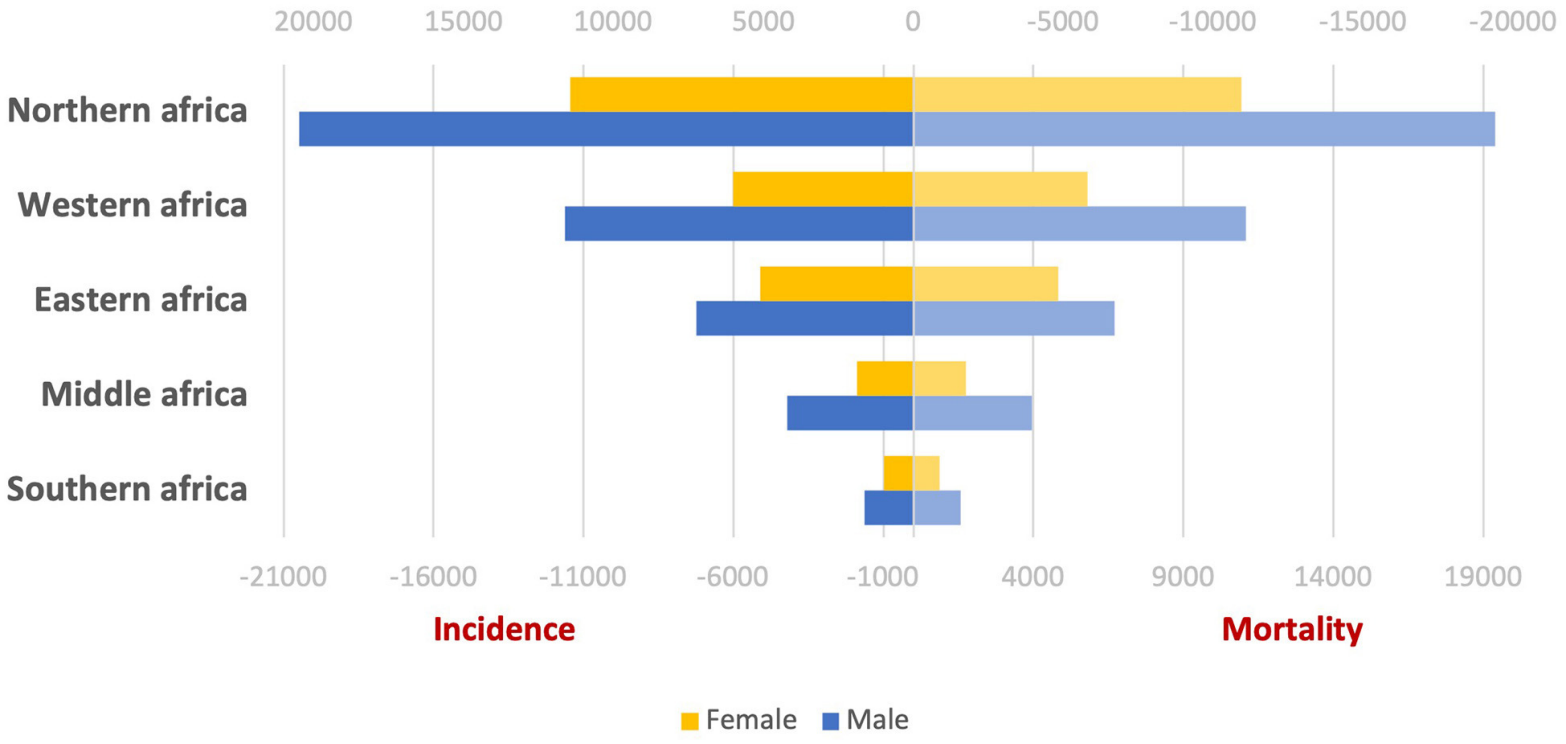
Incidence and mortality of liver cancer in different regions of Africa.

According to the latest Global Cancer Statistics (GLOBOCAN 2020), the incidence and mortality of liver cancer cases in Africa represent 7.8 and 8.1% of the global cases, respectively. The liver cancer incidence and mortality statistics in different African areas for males and females were picked up and represented in Figure 1. African areas were arranged descendingly according to the number of affected cases.

In 2020, SSA had the fourth-highest number of diagnosed PLC cases worldwide after South-Eastern Asia, South Central Asia, and North America, with more than 38,000 new cases of PLC, 77% of them are HCCs (5).

## Risk Factors

The main risk factors for HCC are chronic infection with hepatitis B virus (HBV) or hepatitis C virus (HCV), aflatoxin-contaminated foods, heavy alcohol consumption, obesity, type 2 diabetes, and smoking (4). There is heterogeneity in the distribution of these risk factors between low/middle-income countries and high-income countries. According to the International Agency for Research in Cancer (IARC) 2020, the age-standardized (for all ages, standardized to the world population) incidence rate of liver cancer in low and medium human development index countries per 100,000 persons is 6.9, and 3.2 in male and female, respectively. Meanwhile, the age-standardized mortality rate is 6.7 and 3.1 in males and females, respectively (2). Its incidence is higher with chronic liver diseases, especially viral hepatitis, alcoholic hepatitis or non-alcoholic fatty liver disease (NAFLD) (6).

HCC incidence is 46,000/year in SSA, with age-standardized occurrences as high as 41.2/100,000 each year. Mozambique has the highest rate of HCC in the world. The tumor strikes rural and, to a lesser extent, young urban black Africans and affects more men than women. Cirrhosis coexists with HCC in roughly 60% of patients. The tumor is not only frequent in Black Africans but also has a particularly bad prognosis, with roughly 93% of patients dying within a year after diagnosis (7).

## HBV

The WHO projected in 2015 that 257 million people worldwide had chronic HBV infection, with the African and Western Pacific regions bearing the brunt of the burden with a 15–40% lifetime risk of cirrhosis, liver failure, or HCC (7). Over 50–60% of the HCC cases in SAA are HBsAg positive (8, 9), and it is thought that another 20–50% of patients had previous chronic HBV infection or occult HBV infection (10). The actual number of HCC patients due to HBV past infection is probably underestimated and needs better documentation. Meanwhile, a relatively lower prevalence of HBsAg has been reported in East and South Africa (6–8%) (11). Occult HBV was found in 12% of the HCC cases in black South Africans, and was associated with a four-times higher risk of HCC (12).

## HCV

In 2018, 71.1 million people were estimated as worldwide chronic carriers of HCV; approximately 18 millions of them were in Africa (13) and are at risk of HCC development (14). HCV prevalence in SSA and North Africa is estimated at 2.1–3.3% and 2.3–7.7%, respectively (15). Chronic HCV infection has also been identified as the primary cause of HCC in North Africa (particularly Egypt) (16) and in central Africa (Cameroon) (17). Being the country with the highest worldwide prevalence of HCV, the Egyptian Ministry of Health (MOH) launched a large national screening program that intended to screen the whole country citizens (18). All screened subjects with proven HCV infection were referred for engagement in a government-funded treatment program employing direct-acting antiviral (DAA). However, there is still no national campaign for HCC surveillance (16). Given the magnitude of Egypt's HCV and HCC problems, the extensive HCV treatment program could significantly impact the country's HCC figures shortly (19). Similarly, the Rwandan viral hepatitis program began in 2011, and the national hepatitis elimination strategy declared an ambitious goal of treating 90% of people affected by 2024 (13).

## Environmental and Host-Related Factors

Aflatoxin B1 (AFB1) is a major causing factor of HCC in Africa. It is a mycotoxin produced by aspergillus fungus, classified by WHO and IARC as a “class 1” human carcinogen. This fungus grows in warm places on stored grains (such as peanuts and corn) which are the basis of many African traditional meals. Studies reported that the population aflatoxin-related HCC risk is 17% overall, 8.8% in HBsAg negative populations, and 21% in HBsAg positive individuals (20).

Excessive alcohol consumption is a neglected habit in most African countries, so its role in developing HCC in Africa is underestimated (20). However, a recent WHO report noticed an increase in African alcohol consumption (21).

## HIV and Hepatitis Delta

The proportion of cases attributable to HIV and/or hepatitis delta co-infections is poorly analyzed, although these viruses are highly endemic in Africa (20). Furthermore, no available data about an increased risk of HCC in HIV infection. This is consistent with an earlier case-control study from SA. Although it has been suggested that HIV infection accelerates liver damage related to HBV infection, a possible explanation for this finding might be the reduction in the continual inflammation related to immune-mediated clearance of HBV infected hepatocytes. Additionally, many South African HIV-infected patients died earlier in the absence of effective treatment, given the long induction period needed for HCC to develop (12, 22).

## NAFLD/NASH

The global prevalence of NAFLD was estimated to be 23.4, 23.7, 31.8, 24.1, 30.5, and 13.5% in Asia, Europe, Middle East, North America, South America, and Africa, respectively, according to a recent meta-analysis of studies using imaging or liver biopsy for diagnosis (23). NAFLD/NASH is likely to play a role in the increased risk of HCC in SSA, especially given that changing eating habits, sedentary lifestyles, and the widespread use of antiretroviral therapy (ART) for HIV may all have contributed to the rise in adiposity and diabetes seen in SSA between 2000 and 2014 (12).

## Hereditary Hemochromatosis (HH) and Iron Overload

HH is an autosomal recessive disorder that causes iron accumulation in many organs of the body, including the liver (24). This pathophysiologic propensity to iron excess could progress to cirrhosis and, eventually, HCC HH risk estimates for HCC ranged from 200 in early studies to 20 in more recent ones. Although HH is rare among Africans, it has been observed in Cameroonians, South Africans, and African Americans (12).

## HCC in North Africa: a Unique Situation

It has been worth mentioning that the characteristics and risk factors of HCC differ in Egypt from other African countries (16). Additionally, the proportion of patients receiving specific treatment in other African countries appears to be low, and their outcomes were extremely poor.

Yang et al. performed a study that involved data of 2,566 HCC patients from 21 African centers (in Egypt, Nigeria, Ghana, the Ivory Coast, Cameroon, Sudan, Ethiopia, Tanzania, and Uganda) such that 1,251 patients were Egyptians and 1,315 were from the other African countries. The median age at which HCC was diagnosed significantly differs in Egypt than in other African countries (58 years vs. 46 years; *p* < 0.0001). HCV was the leading cause of HCC in Egypt (1,054 patients [84%]), while HBV was the leading cause of HCC in other African countries (597 patients [55%]). Regarding treatment, more patients received treatment for HCC in Egypt than in other African countries (956 [76%] vs. 43 [3%]; *p* < 0.0001). Among patients, 605 patients [48%] in Egypt survived vs. 583 patients [44%] in other African countries; in addition, the median survival was longer in Egypt (2.5 months vs. 10.9 months in other African countries; *p* < 0.0001). Poor survival was independently associated with being from an African country other than Egypt (*p* = 0.01), hepatic encephalopathy (*p* = 0.0004), the diameter of the largest tumor (*p* < 0.0001), log α-fetoprotein (*p* = 0.0188), Eastern Cooperative Oncology Group performance status 3–4 (*p* < 0.0001) and receiving no treatment (*p* < 0.0001) (25).

## Prevention

The fundamental strategy for liver cancer prevention globally is the elimination of viral hepatitis, as HBV and HCV infection account for 56 and 20% of worldwide liver cancer deaths, respectively (26). Improving the population seroprevalence of HBV via public health success in vaccination against HBV and HCV treatment and reducing aflatoxin exposure could dramatically reduce the prevalence of HBV infection and, therefore, the incidence of HCC in high-risk countries (27).

## Discussion

The primary risk factor for HCC development in black South Africans is HBV infection, followed by HCV infection (12). Kew et al. observed a synergistic effect of HBV-HCV co-infection in developing HCC (28). However, this synergism was not found in another analysis because very few participants were infected with both viruses (12).

Minimal data on the survival of African HCC patients is available, which is obtained from cancer registry data collated by the IARC. There is virtually no data about deaths due to decompensated cirrhosis. However, the number of people dying from cirrhosis and those dying from HCC are the same (20). Recently, Dakurah et al. studied the incidence of viral hepatitis-related HCC in Africa between 1980 and 2019. They noticed a steady increase of 1.52% each decade, but this was not statistically significant. They suggested that the incidence could be higher with statistically significant if a better screening and diagnostic capacity for HCC is done in African countries (29). The lack of basic epidemiological data regarding the actual situation of HCC is a massive constraint against better assessment and evaluation, in our opinion.

HCV is the most common cause of HCC in Egypt, while HBV was the most common cause in the other African countries studied. HCC develops at a younger age in Africa than in other parts of the world, with a usually poor prognosis and the death of the majority of those affected during their most productive years. Compared to other African countries, most of which are SSA, Egypt's HCC outcomes are significantly better, possibly due to the early discovery of the disease and the availability of efficient treatment options. To reduce the burden of disease and death from HCC, urgent efforts are needed to develop national and continental policy initiatives. We need to improve the quality of epidemiological data available to motivate engagement against HCC, cancer registries must be established and coordinated throughout Africa. HBV vaccination programs, maternal-to-child and child-to-child transmission prevention measures, universally accessible antiretroviral and antiviral medications, and dietary aflatoxin reduction can all help to lower HCC incidence. Finally, a deeper knowledge of the specific genetic and epigenetic aspects of HCC on the continent will be required to create diagnostics and innovative treatment strategies.

## Author Contributions

ME-K and ME: study design. ME: data collection and writing up of the first draft of the paper. Both authors revised and approved the final version of the manuscript.

## Conflict of Interest

The authors declare that the research was conducted in the absence of any commercial or financial relationships that could be construed as a potential conflict of interest.

## Publisher's Note

All claims expressed in this article are solely those of the authors and do not necessarily represent those of their affiliated organizations, or those of the publisher, the editors and the reviewers. Any product that may be evaluated in this article, or claim that may be made by its manufacturer, is not guaranteed or endorsed by the publisher.
